# Identification of an Alternative Splicing Product of the *Otx2* Gene Expressed in the Neural Retina and Retinal Pigmented Epithelial Cells

**DOI:** 10.1371/journal.pone.0150758

**Published:** 2016-03-17

**Authors:** Christo Kole, Naomi Berdugo, Corinne Da Silva, Najate Aït-Ali, Géraldine Millet-Puel, Delphine Pagan, Frédéric Blond, Laetitia Poidevin, Raymond Ripp, Valérie Fontaine, Patrick Wincker, Donald J. Zack, José-Alain Sahel, Olivier Poch, Thierry Léveillard

**Affiliations:** 1 INSERM, U968, Paris, F-75012, France; 2 Sorbonne Universités, UPMC Univ Paris 06, UMR_S 968, Institut de la Vision, Paris, F-75012, France; 3 CNRS, UMR_7210, Paris, F-75012, France; 4 CEA-Institut de Génomique, GENOSCOPE, Centre National de Séquençage, 2 rue Gaston Crémieux, CP5706, 91057 Evry Cedex, France; 5 Université de Strasbourg CNRS—ICube UMR 7357—LBGI—Faculté de Médecine—4, rue Kirschleger, F- 67085, Strasbourg, France; 6 Université de Strasbourg CNRS—IGBMC UMR7104–1 rue Laurent Fries—BP10142—F67404 Illkirch, France; 7 Wilmer Institute, Johns Hopkins University School of Medicine, Baltimore, Maryland 21287, United States of America; International Centre for Genetic Engineering and Biotechnology, ITALY

## Abstract

To investigate the complexity of alternative splicing in the retina, we sequenced and analyzed a total of 115,706 clones from normalized cDNA libraries from mouse neural retina (66,217) and rat retinal pigmented epithelium (49,489). Based upon clustering the cDNAs and mapping them with their respective genomes, the estimated numbers of genes were 9,134 for the mouse neural retina and 12,050 for the rat retinal pigmented epithelium libraries. This unique collection of retinal of messenger RNAs is maintained and accessible through a web-base server to the whole community of retinal biologists for further functional characterization. The analysis revealed 3,248 and 3,202 alternative splice events for mouse neural retina and rat retinal pigmented epithelium, respectively. We focused on transcription factors involved in vision. Among the six candidates suitable for functional analysis, we selected *Otx2S*, a novel variant of the *Otx2* gene with a deletion within the homeodomain sequence. *Otx2S* is expressed in both the neural retina and retinal pigmented epithelium, and encodes a protein that is targeted to the nucleus. OTX2S exerts transdominant activity on the tyrosinase promoter when tested in the physiological environment of primary RPE cells. By overexpressing OTX2S in primary RPE cells using an adeno associated viral vector, we identified 10 genes whose expression is positively regulated by OTX2S. We find that OTX2S is able to bind to the chromatin at the promoter of the retinal dehydrogenase 10 (*RDH10*) gene.

## Introduction

Alternative pre-mRNA splicing plays an important role in generating protein diversity [1]. This diversity, which ranges from subtle differences in protein activity to the generation of isoforms with antagonistic or distinct functions, plays an important role in increasing biological function of genes [2]. An example of such alternative splicing-mediated bifunctionality is the nucleoredoxin-like 1 gene, *Nxnl1*, which encodes both RdCVFL, a thioredoxin enzyme, and RdCVF, a trophic factor [3–5]. Retinal tissue has high level of alternative splicing [6], and deregulation of this process through mutations in general splicing factors has been associated with retinal diseases [7–9]. For example mutations in the splicing factor gene *PRPF31* can cause *retinitis pigmentosa*, the most prevalent inherited retinal disease triggering photoreceptor degeneration [10,11].

Alternative splicing provides additional complexity to gene regulation [12]. Transcription factors are highly modular in nature, often containing distinct domains for DNA binding, oligomerization, subcellular localization, and transcriptional activation or repression. Transcription factors variants could impact widely on the regulation of genetic programs, providing a mean for fine-tuning biological systems as well as accreting additional functional diversity [13,14]. An example is provided by alternative splicing for *PAX6*, the master gene for the eye development [15,16]. *PAX6* encodes a homeodomain-containing transcription factor of the paired class that recognizes target genes through its paired-type DNA-binding domain. The human *PAX6* gene produces two alternatively spliced isoforms that have distinct structure of their paired domain. The 14 amino acid insertion encoded by an alternatively spliced exon 5a in the N-terminal DNA-binding paired domain modifies the DNA-binding ability of PAX6, and thereby generates a molecular switch that modifies target specificity [17]. Interestingly, *PAX6(5a)* has been proposed to participate in a developmental cascade leading to the formation of the fovea, the central region of the retina enriched in cone photoreceptors. These photoreceptors sustain central visual acuity in primates [18]. In addition to *PAX6*, other transcription factors such as *NR2E3*, *NRL*, *OTX2* and *CRX* are also essential for retinal development [19–21].

In order to evaluate the repertoire of splicing in the retina, we constructed and sequenced two normalized libraries, one from mouse neural retina (NR) and the other from rat retinal pigment epithelium (RPE). These libraries provide an important resource of biological information on gene expression and on splice variants with respectively 3,248 and 3,202 alternative splice events identified. We focused here on alternative splicing of transcription factors because of their role in regulation of developmental programs. Six transcription factor genes containing splice variants suitable for functional characterization were identified. Among 16 clones encoding for the *Orthodenticle homologue of drosophila* (*Otx2*) gene, two have an extra sequence 5’ of exon 2 and correspond to OTX2L, and one has a shorter exon 2 in the 3’ direction never reported previously and was named OTX2S. We demonstrate here that the new variant OTX2S possesses transdominant regulatory activity and is able to bind to the chromatin of the *RDH10* promoter.

## Materials and Methods

### Animals

Care and handling of animals conformed to the rules established by the association for research in vision and ophthalmology. Mice and rats were housed under the light/dark (12/12 h) cycle, 23–25°C room temperature and offered *ad libitum* access to food and water. This study was carried out in strict accordance with the recommendations in the Guide for the Care and Use of Laboratory Animals of the French ministry of research. The protocol was approved by the Committee on the Ethics of Animal Experiments of the University Pierer and Marie Curie and the French ministry of research (Permit Number: APAFIS#1028–2015070211275177). The animal experiments were performed under the following authorization: “Certificat d'autorisation d'expérimenter sur les animaux vertébrés A-75-1863. Préfecture de Police de Paris (November 9^th^ 2011-November 8^th^ 2016)”. The mice were sacrificed by elongation and the rats by CO_2_. The libraries were constructed using C57Bl/6@N mice, age 8 and 35 days (ratio 1/1) and 8 week-old Long-Evans rats. The expression of *Otx2* variants was analyzed in 8 week-old Long-Evans rats. Animals were sacrificed and eyes were removed for neural retina and RPE dissection.

### RNA purification

The neural retina of 8 and 35 aged mice (C57Bl/6@N) were dissected as previously [22]. 800 eyes from 8 weeks old Long-Evans rats opened by a circumferential incision just below the *ora serrata* and the anterior segment and vitreous were discarded. The retina was gently lifted off the eyecup and the eyecup was rinsed in phosphate buffer saline (PBS). The internal side of the eye was exposed and RLT plus buffer (RNeasy mini kit, Qiagen) was placed on top of RPE allowing cell lysis. The RPE layer was collected by up down pipetting and placed in guanidine hydrochloride solution. RNAs were purified using cesium chloride (CsCl_2_) method [23].

### Neural retina and retinal pigment epithelium library construction

The purified RNAs were used to produce oligo dT-oriented cDNAs that were cloned in a CMV vector (pCMV sport6, Invitrogen). The neural retina libraries from 8 and 35 aged mice were mixed in an equal ratio and normalized using a protocol adapted from Soares *et al*. [24]. The normalization process was monitored using *Unc119*, while the RPE library was normalized and evaluated using *EF1a1*. Both normalized libraries were swapped into pEXP-cDNA3.1 by gateway technology (Invitrogen). All clones are maintained as glycerol stocks at -80°C at the Institut de la Vision and labeled with an identification number (**S1A Fig**). Both libraries were sequenced from the 5’ prime end using T7 promoter primer, 5’ TTAATACGACTCACTATAGGG. Furthermore, all clones in both libraries with gene ontology term, transcription, were sequenced also from the 3’ prime end using BGH primer, 5’ CTAGAAGGCACAGTCGAGGC. Finally, 2,593 internal primers were used to sequence the entire insert of the remaining unassembled clones.

### Bioinformatics analysis and database construction

Sequencing of the cDNAs libraries was followed by mapping onto Mus GoldenPath build 37 for mouse and Rattus GoldenPath Rno3.4 for rat, using basic local alignment tool (BLAT). The boundaries of the exons were determined using est2genome [25]. Protein coding sequence (CDS) prediction was performed using vobocds, an algorithm which chooses the best score for the open reading frame (ORF) finder after in *silico* cDNA translation. Genomic clustering was done with EnsEMBL61 annotations using the definition that cDNAs are in the same cluster only if they share at least 100 nucleotides with the respective genomes. Mapping with Ensembl enabled assignment of a gene name and Refseq GeneID for each cluster. In addition, the putative splice variants in the libraries were analyzed by comparing exon positions of the different transcript sequences of a given locus using the alternative splice events detection bioinformatic tool, ASEtrap [26]. The data for all clones were organized in RetinaDB database available through a secured web server (http://kbass.institut-vision.org/KBaSS/dna/dnaform.php). RetinaDB provides additional access to public databases: gene ontology and KEGG pathways (**S1B Fig**).

We designed a protocol to compare our data to the human retinal splice junctions of “high-quality” as described in Farkas et al. [27] (**S2 Fig**). Using the human genome coordinates of the “high-quality” splice junctions from the bed file retrieved on GEO website (GSE40524), we constructed a database composed of the sequences of length 100 bases spanning the human splice junctions. This protocol was applied to the mouse neural retina and rat RPE cDNA libraries using the mouse and rat genomic coordinates respectively to create the databases containing the 100 base splice junction sequences. Mouse and rat splice junctions overlapping 5’ and 3’ UTR regions were excluded. Using blastn, all mouse and rat splice junction sequences were compared to all available human splice junction sequences to identify splice junctions absent in human set. To increase the detection sensitivity, blast parameters were set to: no filtering of the query and word-size: 8. A junction was considered mouse/rat specific if no hit longer than 90 bases with an e-value lower than e-40 was observed.

### Plasmid construction

The tyrosinase promoter reporter construct contains a 2.3 kb fragment from mouse *Tyr* gene between positions -2236 and +63, in reference to the transcriptional start site, cloned in pGL2-Basic vector (Promega) upstream of the firefly luciferase gene (a generous gift of Dr. Ballotti) [28]. Expression vectors containing human *MITF* (NM_000248) in pcDNA3.1/myc-His(-) vector (Invitrogen) is a generous gift of Dr. Esumi [29]. The vector carrying the adeno-associated virus 2 (AAV2) genome and the transgene cassette encoding eGFP under control of a cytomegalovirus (CMV) promoter (pAAV2-CMV-eGFP) is a generous gift of Pr. Bennett [30]. Expression vectors containing rat *Otx2* splice variants from the normalized libraries are identified by their internal identification numbers in retinaDB: LA0ACA144YK13CM1 (*Otx2*), LA0ACA6YL17.CONTIG (*Otx2L*) and LA0ACA63YE20CM1 (*Otx2S*). The plasmid pAAV2-CMV-*Otx2S* was constructed by replacing the eGFP in pAAV2-CMV-eGFP plasmid using NotI (5’) and BamHI (3’). The rat *Otx2S* fragment was amplified from plasmid LA0ACA63YE20CM1 via high fidelity PCR using the forward primer 5’-GTGTCCAGGCGGCCGCAAAAATGATGTCTTATCTAAA and reverse primer 5’-AATCGGATCCCGATATCTCACAAAACCTGGAATTTCCA.

### Production of the AAV2.1 viruses

The AAV2 vectors with transgene cassettes encoding for green fluorescent protein (GFP) or *Otx2S* under control of the CMV promoter were packaged into an AAV1 capsid by triple transfection using CaCl_2_ / HEPES in HEK293 cells as described [31]. The helper plasmid (pHelper) providing the three adenoviral helper genes and the plasmid encoding for the proteins of AAV1 capsid, pLT-RC02, is a generous gift of Dr. Bemelmans. Viral particles were collected from cell lysates produced from triple freezing and thawing of cells and centrifugation to remove cell debris. Viral particles were purified by iodixanol gradient centrifugation as described [32] and stored in PBS, 0.001% pluronic. Titers were determined by real time PCR as described [33].

### Reverse transcription and real-time PCR

The endogenous expression of genes in primary pig RPE cells overexpressing the *Otx2* variants as well as the endogenous expression of *Otx2* splice variants in rat RPE cells were quantified by real time RT-PCR using RNA extracted using CsCl_2_ [23]. First stranded cDNA was synthesized from 1 μg of total RNA with random primers (Promega) and Superscript II reverse transcriptase (Invitrogen) following manufacturer’s instructions. The cDNA were purified by phenol-chloroform extraction and ethanol precipitation and dissolved in 40 μl of 10 mM Tris-HCl pH 8.0, 1 mM EDTA (TE). The sequence of the primers used for expression screening in pig RPE is given in **S1 Table**. To co-amplify the *Otx2* splice variants in the rat RPE, we used primers P1, forward 5’-GGGCTGAGTCTGACCACTTC and P2, reverse 5’- GCTGACGGCACTTAGCTCTT. Touchdown PCR was performed using primers specific to each isoform, *Otx2*: P3, forward 5’-GTGGGCTACCCGGCCACT and P4, reverse 5’-CTGCACCCTGGACTCTGGC; *Otx2L*: P5, forward 5’-CTGGGCTTCTTGTCCTGCAG and reverse P4; *Otx2S*: P3, and P6, reverse 5’-TAGCTCTTCGATTCTTAAACCATACCTC, starting from 68°C and decreasing 1°C every two cycles to reach 58°C, and followed by 10 additional cycles. Individual quantification of the expression of *Otx2* splice variants was performed by real time PCR, using specific primers P3, P4, P5 and P6. The expression of *Otx2* splice variants was normalized to that of *Actb*: forward 5’-CCTGGGTATGGAATCCTGTG and reverse 5’-CTTCTGCATCCTGTCAGCAA and expressed as 2^-ΔCt^. Primers used for the quantification *Rho*: forward 5’-ACGTCACCGTACAGCACAAG and reverse 5’-TGGGCCCAAAGACAAAGTAG and for *Rpe65*: forward 5’-CCGGATTCTTACCCATCTGA and reverse 5’-AGTCCATGGAAGGTCACAGG. Real-time PCR was performed using Power SYBR Green PCR Master Mix (Invitrogen) and Applied Biosystems 7500 Fast Real-Time PCR System following manufacturer’s instructions. For gene expression screening, the expression of each gene was normalized by the expression of pig *GAPDH* and the expression of each gene was calculated as the ΔΔCt of cells infected with AAV2.1-*Otx2v* with that in AAV2.1-GFP (control) infected cells and shown as 2^- ΔΔCt^.

### Retinal detachment

Retinal detachment was induced in wild-type rats (rdy+/+) aged 2 months by injecting under visual inspection 50 to 200 μl of Hank's Balanced Salt Solution (HBSS) containing 4% chondroitin sulfate and 3% sodium hyaluronate. The animals were sacrificed 8 days after surgery and the neural retina was purified to proceed to quantitative RT-PCR analysis.

### Cell culture

GripTite 293 MSR cells (HEK293 cell line, Invitrogen) were cultured as described [34]. Transfection was carried out using lipofectamine 2000 (Invitrogen) following manufacturer’s instructions.

### Pig retinal pigment epithelium primary culture

Pig eyes were collected from pigs of three breedings: Piétrain, Large white and Landrace and were obtained from a slaughterhouse (Abattoir Guy Harang, Houdan, France). The eyes were disinfected with 95% ethanol and dissected to remove cornea, lens, and neural retina. RPE / choroid, eyecups were washed twice with PBS and filled with trypsin–EDTA 0.25% up to two third of the eyecups for digestion at 37°C for 1 h and 40 min. RPE cells were collected by gentle pipetting and transferred into DMEM containing 20% fetal bovine serum (FBS) and 10 μg/ml gentamicin. The cells from 11 eyes were pooled and plated into a 10 cm^2^ dish in the same medium. The culture medium was changed on day 1 and day 4. By days 5–6, the cultures became confluent and showed a cobblestone like appearance typical of RPE cells. For transfection, RPE primary cells cultured for 1 week were treated with trypsin–EDTA 0.05% and seeded in 48-well plates at 10^5^ cells/well in 250 μl of DMEM containing 10% FBS. The next day, transfection was carried out using lipofectamine plus (Invitrogen). Transfection of 30–40% efficiency was estimated using a plasmid encoding for GFP (**S3 Fig**).

### Transient transfection

Gene reporter studies were carried out utilizing dual luciferase assays (Promega) using GripTite 293 MSR (HEK293) cells (Invitrogen) or pig RPE primary cells. Dual luciferase assay using HEK293 cells were performed as described [34]. For transfection, the total quantity of DNA, the volume of reagents and medium added to each well were equalized. For testing candidate transcription factors, a reaction mixture containing 2 μg of DNA containing 1 μg of firefly luciferase construct [(pGL2-Basic) mouse tyrosinase promoter upstream of firefly luciferase] and 1 ng of pRL-TK *Renilla* luciferase reporter vector (Promega) for normalization was used. The amount of DNA encoding for *Otx2* splice variants used, was 0 to 1 μg of plasmids and the total amount was adjusted to 1 μg by adding empty vector, pcDNA3.1 (Invitrogen). We divided the transfection reaction in quadruplicates of 50 μl in four wells of culture. To assay for synergy, 0.2 and 0.5 μg of *Otx2* splice variants were mixed with 0.2 μg of plasmid encoding for the human MITF, the total amount was adjusted to 1 μg by adding pcDNA3.1 plasmid to which was added 1 μg of firefly luciferase construct (mouse tyrosinase promoter), and 1 ng of internal control plasmid pRL-TK. The experiment was used to perform quadruplicates. To assay for transdominant negative effect of *Otx2S*, 0.2, 0.4 and 0.6 μg of *Otx2S* splice variant was mixed with 0.2 μg of *Otx2* or *Otx2L* plasmid, the total amount was adjusted to 1 μg by adding pcDNA3.1 to which was added 1 μg of firefly luciferase plasmid (mouse tyrosinase promoter) and 1 ng of internal control plasmid pRL-TK. The experiment was used to perform triplicate measurements. The cells were lysed with 100 μl of passive lysis buffer (Promega) after 48 h of culture. To measure luciferase activity using dual luciferase assay, 50 μl of firefly luciferase reagent and 50 μl of *Renilla* luciferase reagent were subsequently injected to 20 μl of cell lysate in each well and recorded for 10 s using a luminometer (MicroLumat Plus LB 96V, Berthold) after of an initial delay of 2 sec. Each experiment was repeated three times. Firefly luciferase activity was normalized by *Renilla* luciferase activity, and relative luciferase activity was calculated as the ratio of the normalized luciferase activity with the expression vectors to that with pcDNA3.1. The activity of the thymidine kinase (TK) promoter was only marginally affected by OTX2 or MITF (**S4 Fig**).

### In vitro transduction

For *in vitro* infection of primary pig RPE, cells were seeded in 12-well plates, 12x10^6^ cells/well in DMEM containing 10% FBS. The next day, cells were washed with PBS and 300 μl of DMEM without serum, and 2.6x10^11^ viral particles (AAV2.1-GFP or AAV2.1-*Otx2S*) were added. After 5 h incubation, the medium was adjusted to 10% FBS with 10 μg/ml gentamicin. The cells were incubated for two weeks at 37°C, 5% CO_2_. The medium was changed every 4 days. For *in vitro* infection of HEK293, 6x10^5^ cells were seeded in each well of 12-well plates (500 μl) and infected with 9x10^10^ viral particles (AAV2.1-GFP, AAV2.1-*Otx2* or AAV2.1-*Otx2S*). After 5 h incubation, the medium was adjusted to 10% FBS with 10 μg/ml gentamicin. The cells were incubated for 11 days at 37°C, 5% CO_2_.

### Western blotting and immunohistochemistry

Western blotting protocol is described in [35]. 48 h after transfection, HEK293 cells were solubilized in lysis buffer [50 mM Tris-HCl-pH 7.5, 1 mM EDTA, 1 mM DTT, 50 mg/ml TLCK (Sigma), 1x protease inhibitors (Sigma), 10 μg/ml Triton X-100] followed by sonication. The antibodies used are the following: anti-OTX2 (ab9566, Millipore 1/1,500), anti-ACTB (1/500). For immunocytochemistry, HEK293 cells were seeded in glass slides and fixed 10 min with 4% paraformaldehyde (PFA) 48 h after transfection. Non specific binding sites were blocked by incubation of slides 1 h at room temperature with bovine serum albumin (BSA) in PBS. OTX2 splice variants were detected using anti-OTX2 (ab9566, Millipore 1/4,500). Immunostained cells were visualized using the Alexa Fluor 594 goat anti-rabbit IgG (A-11012, Invitrogen 1/1,000) and cell nuclei marker, DAPI (1/500) in 1xPBS, 3% BSA. After washing slides were mounted with fluoromount-G (0100–01, SouthernBiotech) and imaged using a microscope (Leica).

### Chromatin immunoprecipitation

Chromatin Immunoprecipitation (ChIP) was performed as described previously [36]. Briefly, transduced HEK293 cells were cross-linked with ice-cold 4% formaldehyde in PBS for 30 min, rinsed in PBS, and sonicated (Vibra Cell) in lysis buffer [1% SDS, 10 mM EDTA, 50 mM Tris-HCl pH 8.0] and protease inhibitors (Sigma) to an average DNA size of 800 bp. The sonicated sample was centrifuged at 15,000 rpm for 10 min at 4°C and the supernatant was pre-cleared with G-sepharose beads (PI-20399, Ficher) for 1 h at room temperature (RT). Aliquots of 100 μl was diluted to 1.5 ml with dilution buffer (1% Triton X-100, 2 mM EDTA, 150 mM NaCl, 20 mM Tris-HCl pH 8.0) and subdivided in three reactions that were incubated for 1 h at RT with 1) no-antibody 2) 2.5 μg of anti-rabbit antibodies (111-035-045, Jackson lab) antibody 3) anti-OTX2 antibodies (ab9566, Millipore). Samples were centrifuged at 15,000 rpm for 10 min at 20°C and the supernatant was mixed with 15 μl of protein G-sepharose beads, 150 μg ultrapure salmon sperm DNA (15632–011, Invitrogen) and 150 μg yeast tRNA, (15401–011, Invitrogen) and incubated for 1 h and 30 min at RT. Precipitates were washed sequentially for 10 min at RT with TSEI (0.1% SDS; 1% Triton X-100; 2 mM EDTA; 20 mM Tris-HCl pH 8.0; 150 mM NaCl), 4 times with TSEII (0.1% SDS; 1% Triton X-100; 2 mM EDTA; 20 mM Tris-HCl pH 8.0; 500 mM NaCl), once with buffer III (0.25 M LiCl pH 8.0; 1% nonidet P-40; 1% deoxycholate; 1 mM EDTA; 10 mM Tris-HCl pH 8.0), and finally three times with TE buffer. Samples were eluted and cross-links cleared by overnight incubation at 65°C in 100 μl of elution buffer (1% SDS; 0.1 M NaHCO_3_). DNA fragments were purified by phenol-chloroform extraction and resuspended in 70 μl of TE buffer. PCR was used to amplify 2 μl of the immunoprecipated material. PCR reaction was performed in 25 μl for 95°C 15 min, 35 cycles (94°C/15 sec /58°C 1 min /72°C 1 min) and 72°C 10 min. The primers used were designed to amplify fragments into the promoter genes: *RDH10*: forward 5’-CGGGTAAAACTTGTTTGAAG; reverse 5’-CATGCTGGGATTTGTAGTTCG.

## Results

### Construction of neural retina and retinal pigment epithelium libraries

In order to evaluate the repertoire of splice variants in the retina, we constructed two normalized libraries, one from mouse neural retina (NR) and the other from rat RPE. The normalized NR library was constructed using a mixture of two non normalized NR libraries, one from post natal (PN) 8 mouse retina and the other from PN35 mouse retina. Before normalization, the PN8 library had 46.3 x 10^6^ colony forming unit (cfu), a mean insert size of 1.8 kb, with 91.7% of the clones being recombinant. The PN35 library had 7.8 x10^6^ cfu, a mean insert size of 1.7 kb, with 87.5% of the clones being recombinant. After normalization the NR library, made by mixing the two libraries at a 1/1 ratio, had 4.94 x 10^6^ cfu with a mean insert size of 1.8 kb. The RPE library (6.0 x 10^6^ cfu) from 8 weeks rats had a mean insert size of 2.1 kb, with greater than 99.9% of the clones being recombinant. The normalization was estimated by the reduction of clones encoding the unc-119 homolog of *C*. *elegans* (*Unc119*) and elongation factor 1-alpha 1 (*Eef1a1*) genes for mouse and rat, respectively (**Table 1**). The analysis of the normalized libraries shows that they are of optimal quality for the objective since mean insert size that is higher than most cDNA libraries [37,38].

**Table 1:** Normalization of mice and rat libraries.

	Gene used fornormalization	Fold drop	Number of clones (cfu)	Average insert size
**8 and 35 days-old mice**	*Unc119*	106	4.94 x 10^6^	1.8 kb
**8 weeks-old rats**	*EF1a1*	30	6.00 x 10^6^	2.1 kb

### Identification of alternative splice variants

66,217 and 49,489 expressed sequence tags (ESTs) for mouse and rat, respectively, were obtained by 5’ primer sequencing. The ESTs were assembled into contigs using the phrap program (http://phrap.org/phredphrap/phrap.html): two EST were considered to come from the same gene if the overlap was significant according to the scores obtained. Based upon clustering the cDNAs and mapping them with their respective genomes, the estimated numbers of genes (corresponding to clusters in the database) were 9,134 for the NR and 12,050 for the RPE libraries. The genomic clustering for mouse NR and rat RPE libraries covered 23% and 32.5% of the predicted EnsEMBL61 clusters, respectively. The existence of donor-acceptor consensus splice sites was analyzed using ASEtrap [26]. This resulted in detection of 3,248 and 3,202 alternative splice events for a total of 10,457 and 5,536 cDNA clones for the mouse and rat libraries, respectively.

### Identification of splice variant candidates

A secured web server called RetinaDB (http://kbass.institut-vision.org/KBaSS/dna/dnaform.php) was created in order to organize the data. All clones are maintained as glycerol stocks at -80°C at the Institut de la Vision. Users can search using gene name, sequence identifier, cluster identifier, gene description, by nucleic or amino acids sequence using BLAST and by gene ontology terms. RetinaDB is linking the collection of clones to external hyperlinks, NCBI and UCSC genome browser and to localization of the clone within the 96 or 384 well-plate collection (**S1 Fig**). We evaluated that resource by comparing it to the work of Farkas et al. [27] (**S2 Fig**). To identify the mouse/rat splice junctions that have no equivalent in the extended set of human splice junctions, we compared the human library containing 351,547 high-quality splice junctions to the mouse neural retina and rat RPE libraries containing 30,493 and 36,762 splices junctions respectively. In total, 3,833 (12.6%) and 3,927 (10.7%) splice junctions were specific to the mouse neural retina and the rat RPE respectively.

**Fig 1:**
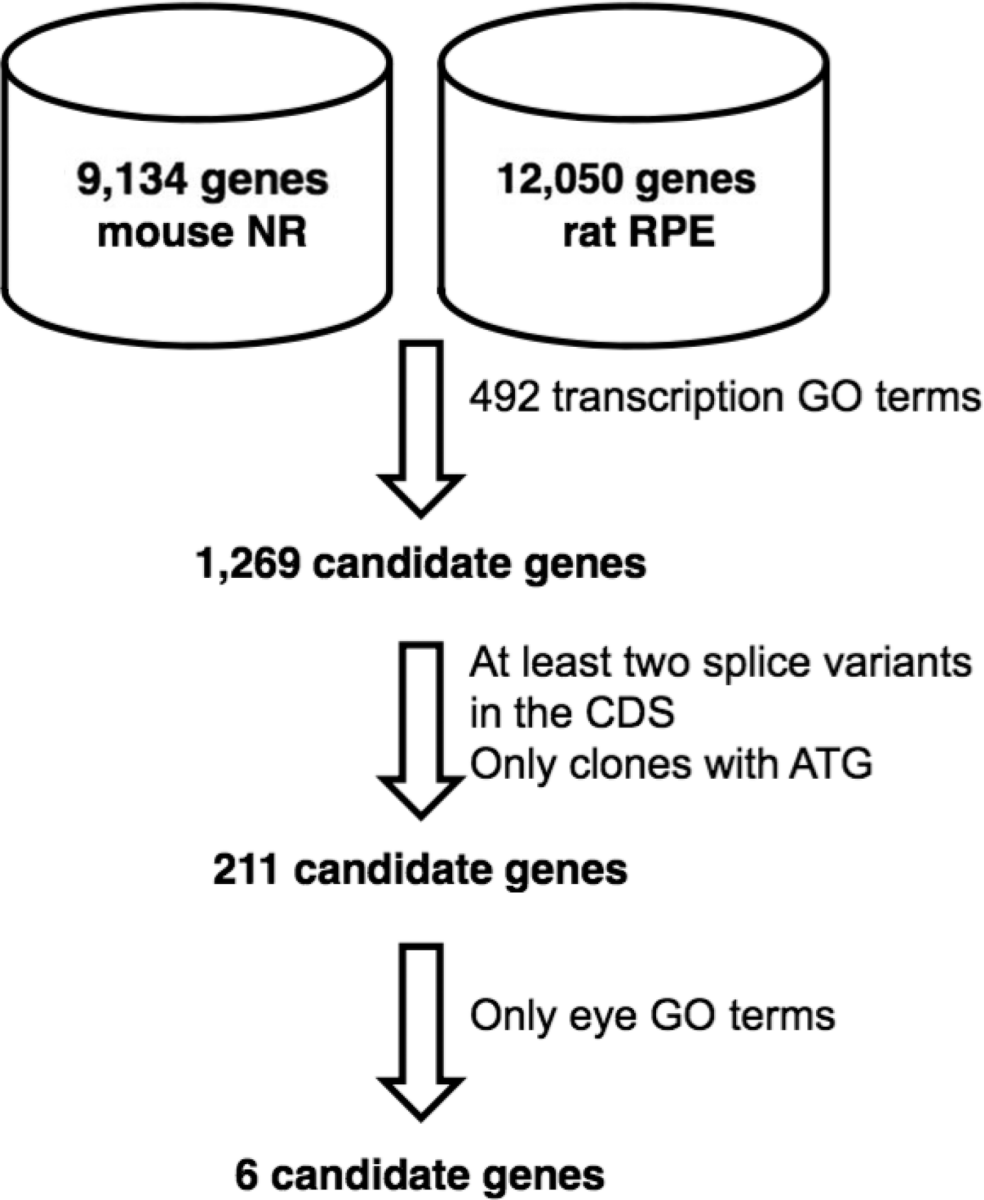
Method used to obtain genes containing splicing variants and the GO term transcription and eye in their ontology.

We focused on transcription factors since they represent major regulators of biological processes. A total of 7,171 clones from the NR and RPE libraries are described by the general gene ontology (GO) term transcription and were identified and sequenced from their 3’ ends, leading to 4,559 pairwise assembled clones. 2,593 internal sequences provided an additional 1,204 pairwise assembled clones for a total of 5,763 fully sequenced clones for a total of 492 GO terms including the word transcription. The redundancy was removed leading to 1,269 candidate genes among the 21,184 mouse (9,134) and rat (12,050) genes in the database (6%). We then selected genes having at least 2 splice variants within the protein sequence and for which at least two representative clones include the sequence translation initiation for functional characterization. This approach reduced the list to 211 candidate transcription factors. Finally, we restricted the list to those with GO terms containing the word eye. This additional filter led to the identification of splice variants in six transcription factor genes (*Meis2*, *Otx2*, *Rax*, *Ring1*, *Rorb* and *Vax2*) related to vision (**Fig 1**and **Table 2**).

**Table 2:** Splicing variant identification in six candidate genes. The table summarizes the splice variants found in the mouse/rat libraries with the identification number for each new splice variant. Splicing process for each compared to the reference sequence. The alignments are based on UCSC browser.

Gene	Specie	Representative clone ID	IntID	Splicing process	ATG	CDS	GenBank	Uniprot
***Meis2***	Mouse	LA0AAA56YF23.CONTIG	1a	-	-#	-	U68383.1*	P97367.3
	Mouse	LA0AAA12YB24.CONTIG	1b	Exon skipping	+	+	New	-
***Otx2***	Rat	LA0ACA23YG03.CONTIG	2a	-	+	-	NM_001100566.1*	F2Z3S7
	Rat	LA0ACA6YL17.CONTIG	2b	Alternate 3’/5’	+	+	New	-
	Rat	LA0ACA63YE20CM1	2c	Alternate 3’/5’	+	+	New	-
***Rax***	Mouse	LA0AAA88YP21.CONTIG	3a	-	+	-	BC058757.1*	O35602
	Mouse	LA0AAA5YJ22.CONTIG	3b	Alternate 3’/5’	-	+	New	-
	Mouse	LA0AAA89YO03.CONTIG	3c	Alternate 3’/5’	+	+	New	-
	Mouse	LA0AAA80YH01.CONTIG	3d	Exon skipping	+	+	New	-
	Mouse	3030000051432591	3e	Exon skipping	+	+	New	-
***Ring1***	Mouse	LA0AAA124YH09.CONTIG	4a	-	-	-	BC009070*	O35730-2
	Mouse	LA0AAA14YL18.CONTIG	4b	Intron retention	-	+	New	-
	Mouse	LA0AAA34YG11.CONTIG	4c	Exon skipping	+	+	New	-
	Mouse	LA0AAA95YP19.CONTIG	4d	Intron retention	-	+	New	-
***Rorb***	Mouse	3030000046806503	5a	-	-	-	DQ779924*	Q8R1B8-2
	Mouse	LA0AAA121YD06.CONTIG	5b	-	+	+	DQ779923	Q8R1B8-1
	Mouse	LA0AAA37YI10.CONTIG	5c	Exon skipping	+	+	BC024842.1	-
***Vax2***	Mouse	LA0AAA100YK05.CONTIG	6a	Exon skipping	+	-	Y17792.1	Q14B19
	Mouse	LA0AAA124YD10.CONTIG	6b	-	+	+	New	-

* Reference sequence.

# Several spliced variants with an initiation codon were initially indentified but none correspond to the reference sequence.

### *OTX2* splice variant expression in retinal pigment epithelium and neural retina

Within the six candidate genes, LA0AAA12YB24.CONTIG, corresponding to the internal ID 1b (**Table 2**), encoding for a variant of *Meis2*, lacks coding exon 8, 9 and 10 compared to the longer isoform P97367.3. The clone 2c, encoding for *Otx2*, uses an alternate 5’ splice site resulting in a smaller exon 2 in the coding region, while 2b uses an alternate 3’ splice site resulting in a longer exon 2 in the coding region, compared to F2Z3S7 form. The clone 3c encoding for a variant of *Rax*, has a shorter exon 2 resulting in an alternate 5’ splice site and a shorter exon 3, which in turn results in an alternate 3’ splice site compared to the larger form, O35602. The clone 3d lacks coding for exon 2 resulting in exon skipping, 3b uses an alternate 3’ splice site resulting in a shorter exon 3, encoding for shorter isoforms of RAX, while 3e has an extra exon 4 compared to the reference RNA sequence encoding for the prototypic RAX protein, O35602. The clone 4c, encoding for a variant of the *Ring* gene, has a shorter exon 4 in the coding region, 4b has a larger exon 4 and 4d, a larger exon 5 compared to the reference RING gene encoding O35730-2. The clone 5c has an extra exon 2 compared to the ROR beta (RORB) isoform, Q8R1B8-2. Finally, the *Vax2* clone 6b has an extra exon 3 compared to the reference VAX2 protein, Q14B19.

**Fig 2:**
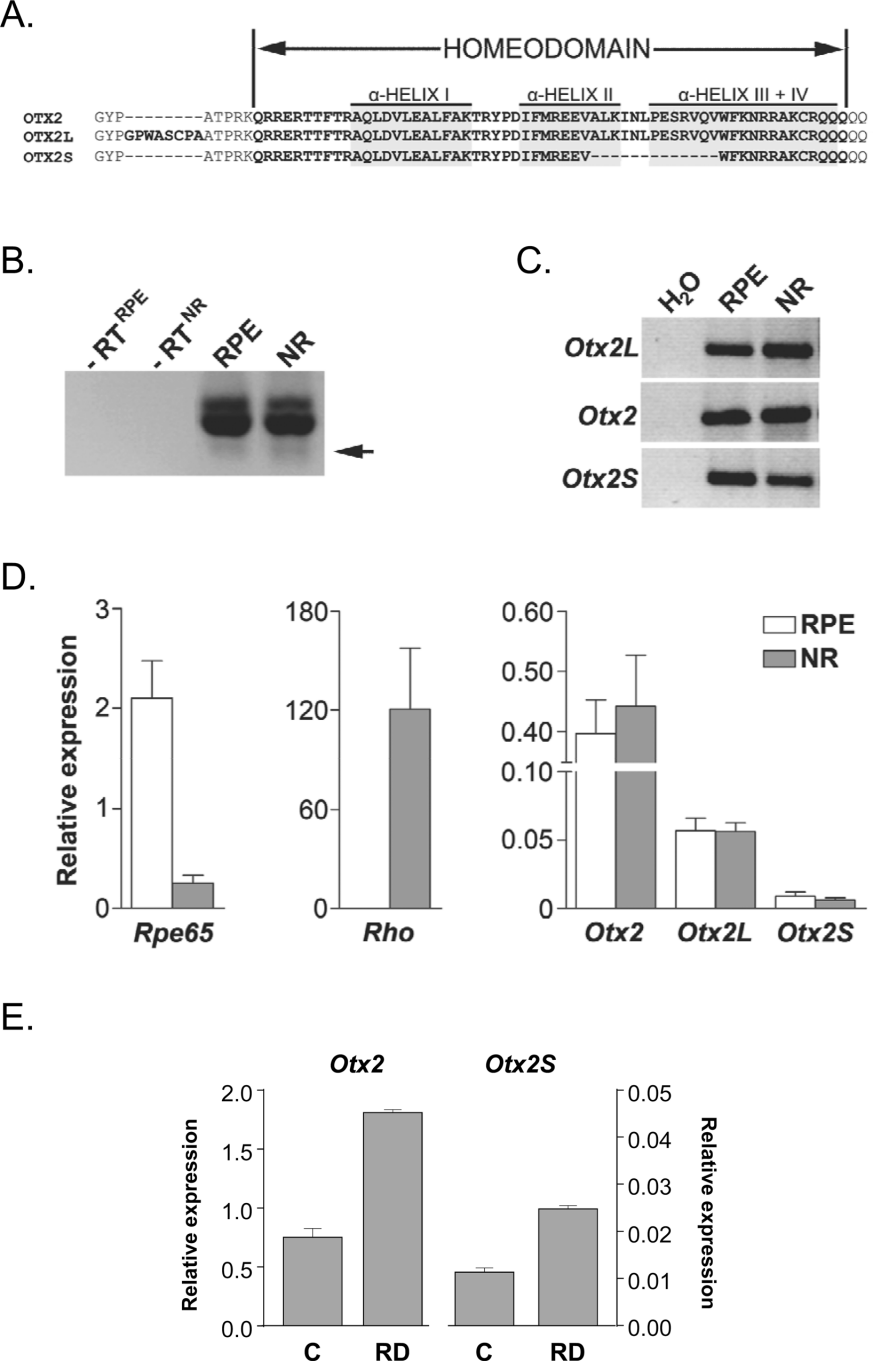
Analysis of the protein sequence and the expression of *Otx2* splicing variants in retinal pigment epithelium and neural retina. (A) Sequence alignment of the protein region corresponding to the homeodomain of *Otx2* splicing variants found in the rat RPE library. (B, C, D) RT-PCR analysis was performed using the cDNA from RPE and NR of Long Evans rats. B) Primers amplifying all *Otx2* splice variants (*Otx2*, *Otx2L* and *Otx2S*: 247, 271 and 208 bp respectively). The RT-PCR product corresponding to *Otx2S* was validated by sequencing after gel extraction. (C) Touchdown PCR analysis using isoform-specific primers for the *Otx2*, *Otx2L* and *Otx2S*. (D) The relative expression of *Otx2* splicing variants was determined using specific primers. No significant contamination between the two tissues was observed by *Rpe65* and *Rho* expression. (E) Quantitative RT-PCR analysis of *Otx2* and *Otx2S* expression in a rat model of retinal detachment. The relative expression is expressed as 2^-ΔCt^. *Actb* was used as the internal control for normalization.

Most of the transcription factors candidates have a role in retinal development [39–42]. For example, *Rorb* knockout mouse has abnormal photoreceptors [43] and *Vax2* counteracts the effect of *Mitf* on RPE differentiation [44]. *Otx2* plays an important role in the development of the eye [45] and its deletion in adulthood leads to photoreceptor degeneration [21,46]. We focused on the functional characterization of the novel variant OTX2S. *Otx2* gene is organized into five exons, only three are translated, so other *Otx2* non-coding variants were also found [47]. The two *Otx2* alternatively spliced variants encode for variant OTX2 proteins (**Fig 2A**). OTX2L corresponding to clone 2b encodes for a protein with a larger exon 2 has an octapeptide GPWASCPA inserted 5 amino acids upstream of the homeodomain AA 38–97 [48]. The second variant protein corresponding to clone 2c was named OTX2S, a novel *Otx2* splice variant. OTX2S has a deletion of 13 amino acids in the α-helices II and III+IV in OTX2. Since the process of normalization results in the reduction of redundancy of a library without affecting its diversity, it is impossible to infer the relative expression of *Otx2*, *Otx2L* and *Otx2S* from the proportion of clones identified by sequencing. In order to address experimentally this question, RNA was prepared from the neural retina (NR) and from the RPE. Three groups of 7 animals were used in order to average the inter-individual variation by pooling 7 animals and to permit statistical analysis with the three groups. The animals were sacrificed at the same time of the day to avoid any difference in gene expression that would be the result of an unsuspected effect of the circadian rhythm. Equal amount of RNA (RPE and NR) was used to synthesize cDNA using a random hexanucleotide and oligonucleotide primers were designed to amplify alternatively all variants of the *Otx2* mRNA (coding for OTX2, OTX2L and OTX2S). We detected the expression of the three variants in RPE and NR, even if the expression of *Otx2S* was barely visible (**Fig 2B**). The sequence of the *Otx2S* amplification product was found to be identical to that of the clone LA0ACA63YE20CM1 (clone 2c). We also designed primers using a sequence specific to *Otx2L* mRNA, and an oligonucleotide primer encompassing the novel junction created by the deletion in the *Otx2S* mRNA (**Fig 2C** and **S5 Fig**). These primers were used in quantitative RT-PCR (**Fig 2D**). The expression of *Rpe65* by RPE and of *Rho* by NR validated the absence of major cross contamination of the RNA preparations since these genes are specifically expressed in RPE and NR, respectively. The expression of *Otx2L* was found to represent 19 and 28% of that of *Otx2* and for *Otx2S*, 2.3 and 1.9% for RPE and NR, respectively. We also examined the expression of *Otx2S* in the neural retina of the rat after retinal detachment (**Fig 2E**). We reasoned that the expression of *Otx2S* might be stimulated by this pathophysiological process which triggers in human patients an inflammatory response and photoreceptor degeneration [49]. *Otx2* and *Otx2S* expression increased after this acute insult but the response of the two spliced isoforms could not be distinguished in that model.

### *OTX2* splice variants localize to the nucleus

**Fig 3:**
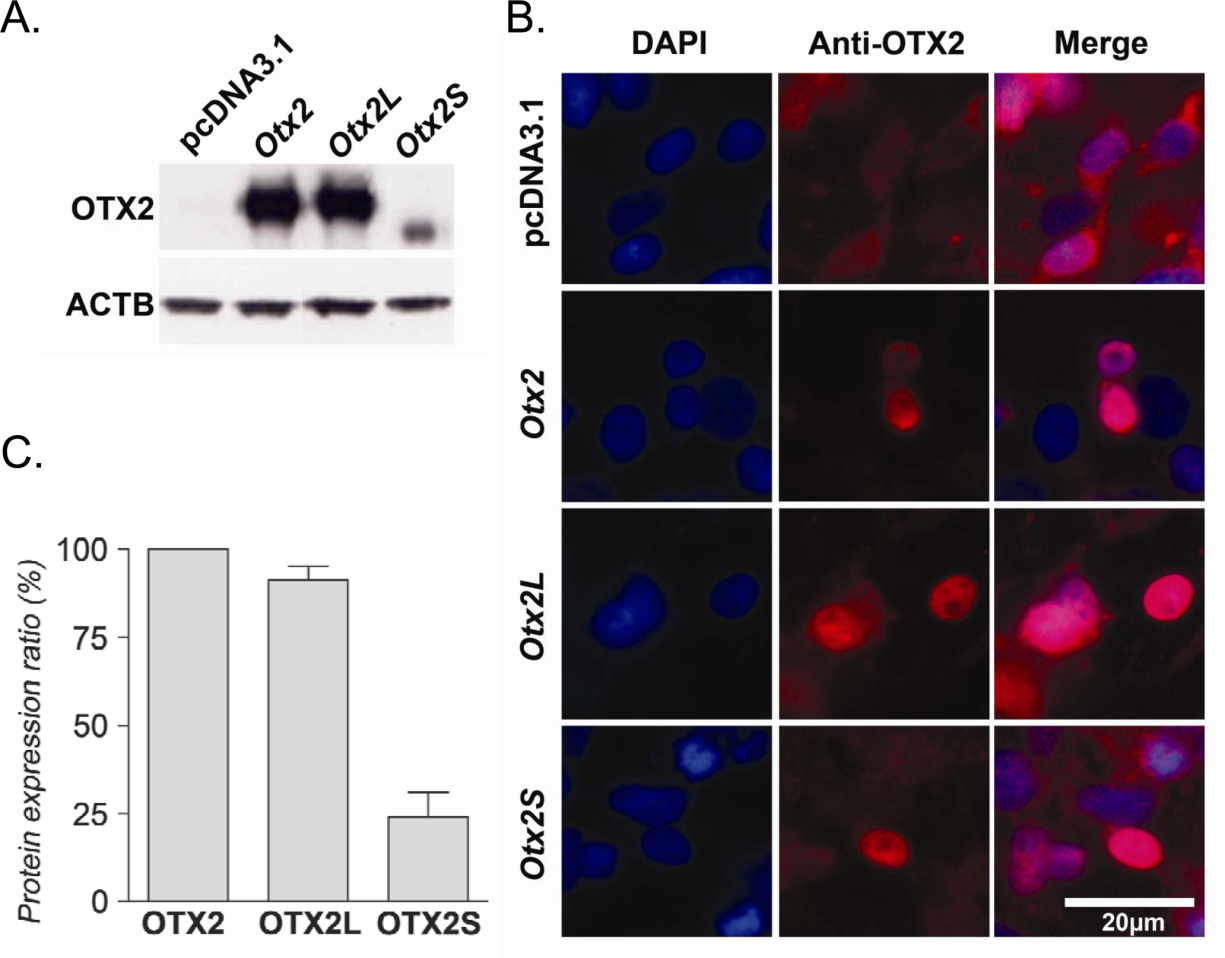
Protein expression and cellular localization of OTX2 variants. (A) Protein expression of OTX2, OTX2L and OTX2S was detected by western blot analysis using polyclonal anti-OTX2 antibodies after transfection of HEK293 cells. HEK293 cells do not express OTX2 protein. The size of the proteins detected is 35, 36 and 33 kDa for OTX2, OTX2L and OTX2S respectively. HEK293 cells transfected with the empty vector, pcDNA3.1, were used as negative control. (B) Quantification of the expression performed using ImageJ. ACTB was used as control for normalization. OTX2L and OTX2S expression is 91% (*P* = 0.0111) and 24% (*P* < 0.0001) respectively of OTX2 expression (100%). Error bars represent standard deviation of the mean. (C) Nuclear localization of the OTX2 isoforms (red) in the nucleus of MSR 293 transfected cells using immunocytochemistry. Blue: DAPI. Scale bar 20 μm.

In order to validate the translation of the OTX2 splice variants, HEK293 cells were transfected by plasmids encoding the rat OTX2 splice variants. Whole cell extracts were prepared in triplicate and analyzed by western blotting using anti-OTX2 rabbit affinity purified polyclonal antibodies. The immunogen of these antibodies was full-length recombinant human OTX2 protein (excluding the first 5 amino acids), and the antibodies most likely recognize all three proteins with similar affinity. The proteins were detected at 35 kDa (OTX2), 36 kDa (OTX2L) and 33 kDa (OTX2S) (**Fig 3A**). OTX2L and OTX2 were produced at similar levels to those observed previously [48]. The deletion of part of the homeodomain of OTX2S likely interferes with its stability, and consequently its expression is lower than the two other isoforms. The quantification of the amount of protein produced after transfection based on three independent transfections shows that OTX2S is expressed at 25% compared to that of OTX2 (**Fig 3B**). We asked whether the deletion in the homeodomain of OTX2S influences its localization within the cell. For this, we performed immunocytochemistry using the same antibodies on HEK293 transfected cells. The results indicate that OTX2, OTX2L and OTX2S are all localized in the nucleus (**Fig 3C**). The weak signal observed with cells transfected with the empty vector pcDNA3 may result from non specific binding of the anti-OTX2 antibody used.

### OTX2S exerts a transdominant negative effect on the tyrosinase promoter

**Fig 4:**
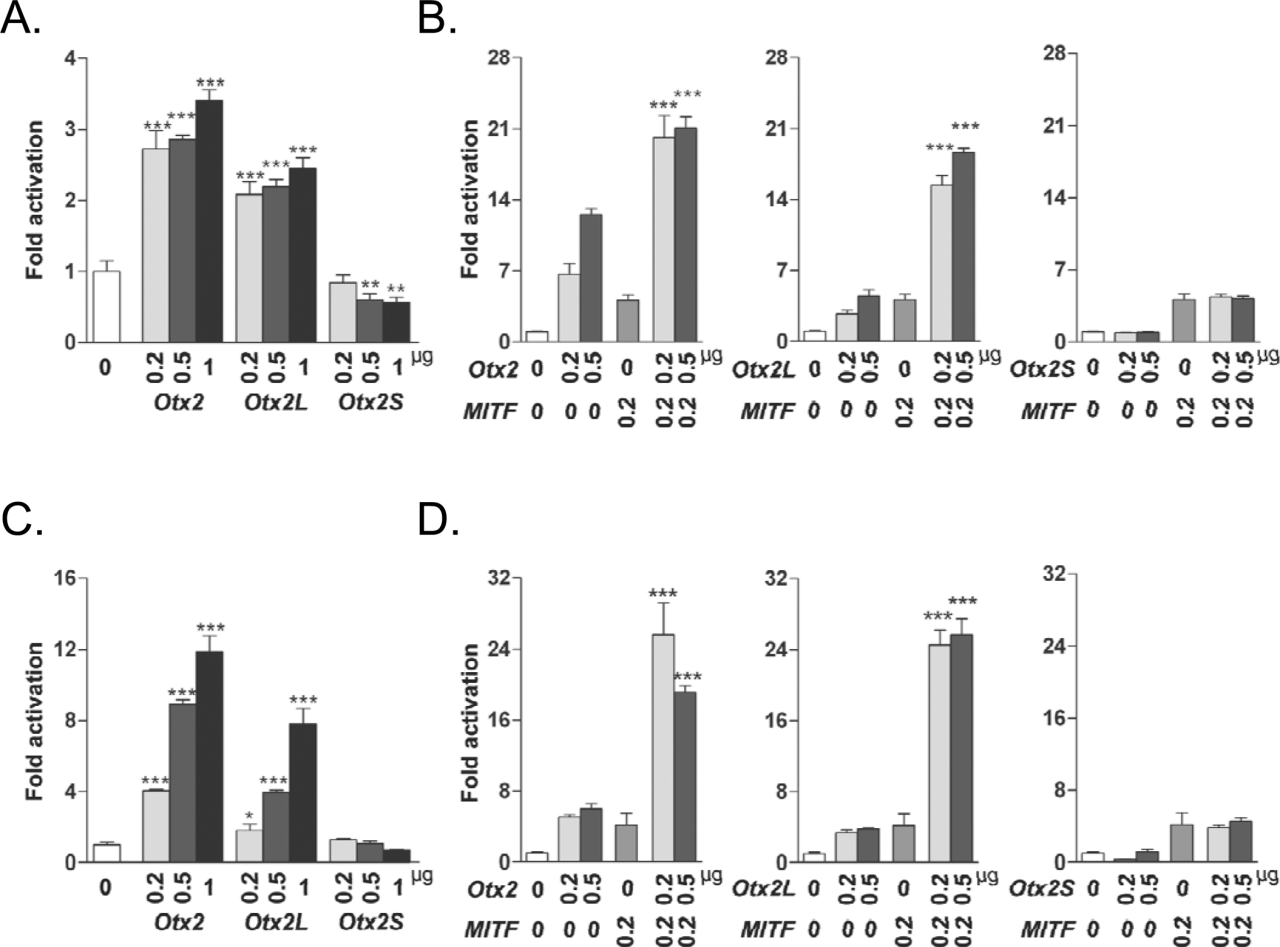
Synergistic activation of the tyrosinase promoter by OTX2 isoforms and MITF. (A) Luciferase assays performed using mouse *Tyr* promoter construct -2226/+63 on HEK293 cells. (B) Luciferase assays performed using the mouse *Tyr* promoter construct on pig primary RPE cells. (C) Luciferase assays performed using mouse *Tyr* promoter construct on HEK293 cells using co-transfection with MITF. (D) Luciferase assays performed using mouse *Tyr* promoter construct on pig primary RPE cells using co-transfection with MITF. Each transfection was repeated three times with quadruplicates. Error bars represent Standard deviation of the mean. * *P* ≤ 0.05, ** *P* ≤ 0.01, *** *P* ≤ 0.001 (Two-way ANOVA Dunnett test).

OTX2 binds to the promoter regions of the MITF target genes dopachrome tautomerase (*DCT*), tyrosinase-related protein 1 (*TYRP1*) and tyrosinase (*TYR*), leading to increased transactivation by itself or in synergy with MITF [50,51]. To test for a possible role of OTX2S in the regulation of the tyrosinase gene, we used 2.2 kb of the mouse tyrosinase promoter cloned upstream of a firefly luciferase reporter [28]. Co-transfections of plasmids encoding the *Otx2* splice variants with the tyrosinase promoter reporter construct were performed in HEK293 cells and in primary pig RPE cell cultures. HEK293 cells were chosen since they do not express endogenous OTX2 (**Fig 3A**) [48]. OTX2L and OTX2 activate the tyrosinase reporter in a dose dependent manner in both the RPE and HEK293 cells, while no activation was observed from OTX2S in either cell type (**Fig 4A** and **4C**). Next we tested for synergistic activity of the OTX2 splice variants with the MITF transcription factor. Co-transfection with human *MITF* and *Otx2* or *Otx2L* resulted in 12-fold increase in both cell types. In contrast, no significant increase in promoter activity was observed with *MITF* and *Otx2S* co-transfection (**Fig 4B** and **4D**). These data suggest that contrary to OTX2S which is inactive when using 0.5 μg of plasmid DNA, the insertion upstream of the homeodomain in the OTX2L protein does not interfere with its activity to bind to and activate the tyrosinase promoter.

**Fig 5:**
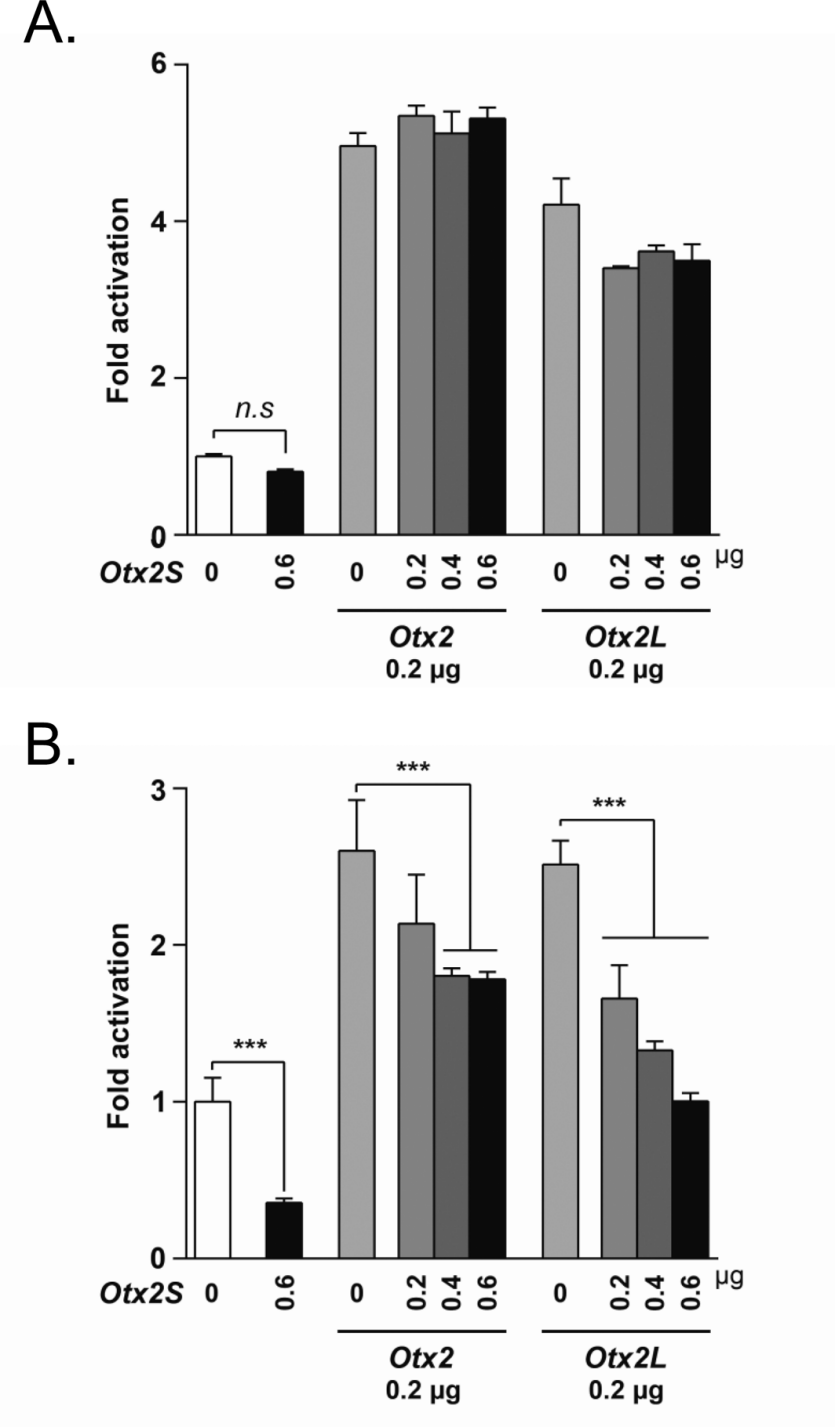
OTX2S exerts a transdominant negative effect on the tyrosinase promoter. (A) The absence of effect of *Otx2S* on tyrosinase promoter in HEK293 co-transfected cells. (B) *Otx2S* has a transdominant activity of tyrosinase promoter on *Otx2* and *Otx2L* activity in primary RPE co-transfected cells. The inhibitory effect of *Otx2S* was found to be higher in *Otx2L* co-transfected assays. Each transfection was repeated three times with quadruplicates wells analyzed. Error bars represent standard deviation of the mean. *** *P* ≤ 0.001 (Two-way ANOVA Dunnett test).

Since *Otx2S* has a truncated homeodomain, which might make it function as a dominant negative activator, we next tested if OTX2S is able to repress the activity of OTX2 on the tyrosinase gene when co-expressed. To test the transcriptional activity of OTX2S on the tyrosinase promoter, we performed co-transfections in HEK293 cells. OTX2S did not modify the activity of OTX2, nor of OTX2L (**Fig 5A**). We also tested primary RPE cells, since they provide a more physiological system to study RPE gene regulation. In this system, increasing amounts of co-transfected *Otx2S* resulted in a dose-depended reduction of promoter activation by OTX2 and OTX2L (**Fig 5B**). The highest dose of OTX2S reduceOXT2-mediated activation by 51.2%. OTX2S also reduce OTX2L activation by 99.7%. No change in the activity of the thymidine kinase promoter driving the *Renilla* luciferase reporter was observed in those conditions (**S4 Fig**) We also noticed that while OTX2S is inactive in HEK293 cells, it reduces the basal level of promoter activity in the primary RPE cells by 64.3% when using 0.6 μg of plasmid, presumably because such cells express endogenous *Otx2*. These results demonstrate that OTX2S, a protein encoded by a novel splice variant of *Otx2*, has transdominant negative activity on the tyrosinase promoter in primary RPE cells, but not in the more artificial setting of HEK293 cells.

### Identification of genes targeted by OTX2S

**Fig 6:**
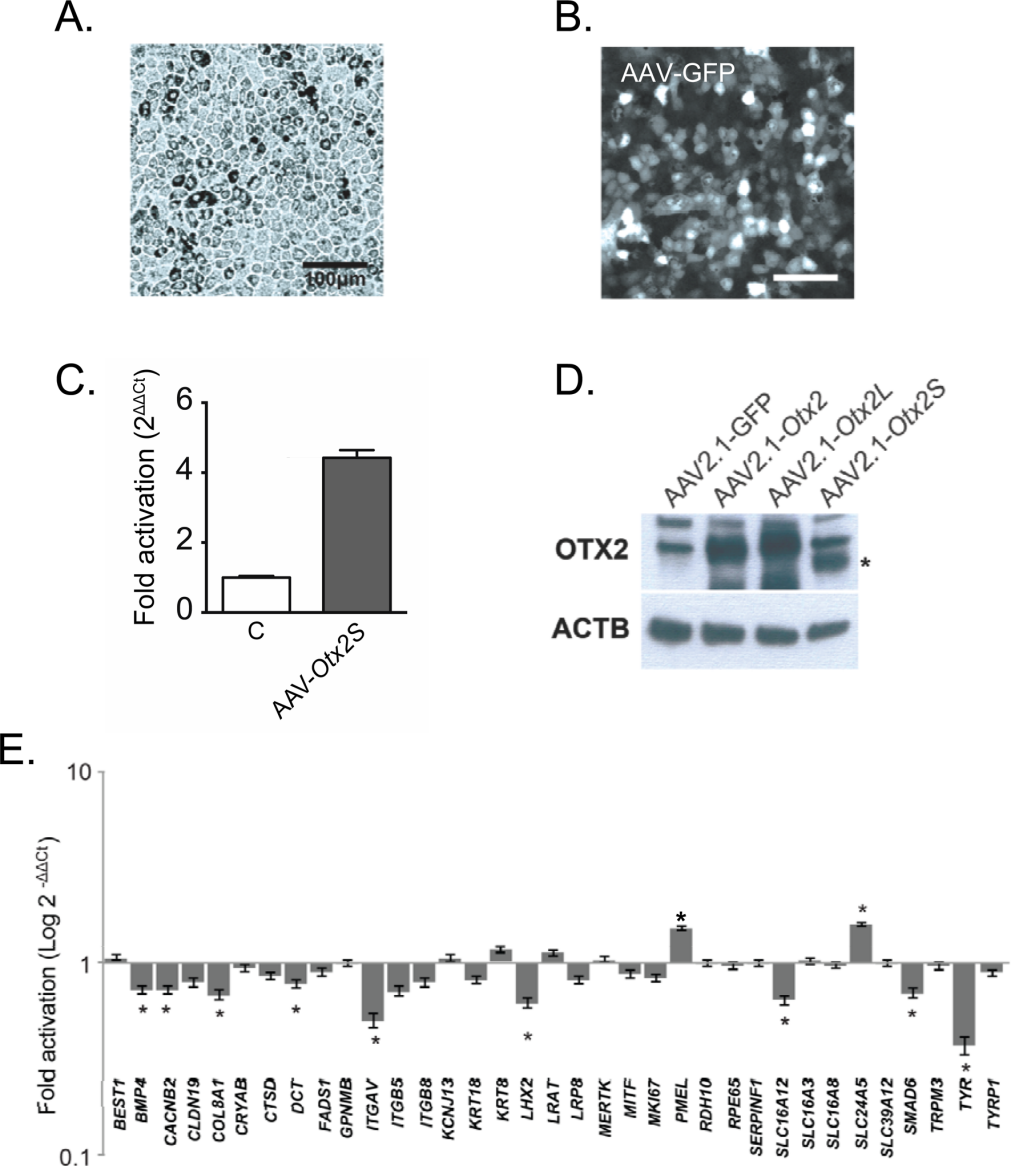
Gene expression profiles in primary RPE cells overexpressing OTX2S splicing variant. (A) Morphology of the primary pig RPE cells before infection. (B) Expression of GFP by RPE cells one week after infection of AAV2.1-GFP. (C) Expression of *Otx2S* in transduced pig primary RPE cells. (D) Western blotting analysis of pig RPE cells infected with recombinant AAV2.1 vectors as indicated. The asterisk points to OTX2S. (E) Quantitative RT-PCR for the indicated genes using mRNA isolated from primary RPE cells infected with adeno associated viruses (AAV2.1) expressing the *Otx2S* splice variant. AAV2.1 expressing the GFP was used as the non treated control (C). GAPDH was used as the internal control for normalization, the expression of which does not change between conditions. The expression of each gene was normalized by the expression in the GFP infected RPE cells and it is expressed as 2-ΔΔCt method. Each experiment was performed in triplicates.*, P < 0.05.

In order to find genes that may be targeted by *Otx2* and be important for mature RPE, we overexpressed the *Otx2* splice variant in pig primary RPE cells using adeno-associated viral (AAV) vectors. The cells display a cobblestone like appearance and pigmentation typical of RPE cells (**Fig 6A**). For transducing RPE cells, we used AAV2.1 particles expressing OTX2S. Indeed, we find that transduction efficiency 12 days AAV2.1 infection of RPE cells is higher that after 3 days by transient transfection (**S3 Fig**). The efficiency of infection was estimated to approach 80% using AAV2.1-GFP as reporter (**Fig 6B**). The expression of the transgene was validated by quantitative RT-PCR 12 days after the transduction (**Fig 6C**). We found that OTX2S protein expression was higher after AAV delivery than transient transfection (**Fig 6D**). We selected 36 potential targets based upon either their specific expression by RPE cells [52] and their possible importance in RPE function [53–56]. We filtered the list for genes whose pig (*Sus scrofa*) orthologs have been annotated. Overexpression OTX2S led to the identification of 10 target genes whose endogenous expression was changed as compared to control RPE infected with AAV2.1-GFP (**Fig 6E**). The modest up-regulation observed for *SLC24A5* and *PMEL* did not allowed us to conclude unambiguously that OTX2S activates the promoter of these two genes. Among 9 genes whose endogenous expression is reduced by OTX2S, we noticed that the highest amplitude (36%) concerns *TYR*, the gene whose promoter is regulated by the transdominant activity of OTX2S (**Table 3**). So, we can conclude that *BMP4*, *CACNB2*, *COL8A1*, *DCT*, *ITGAV*, *LHX2*, *SLC16A12*, *SMAD6* and *TYR* promoters are responsive to the transdominant activity of ectopic OTX2S over the endogenous pig OTX2.

**Table 3:** Relative expression in cultured RPE with AAV-OTX2S versus AAV-GFP control transduced cells.

Gene symbol	Gene name	NCBI accession N°	Relative expression (2^-ΔΔCt^)	*P* value
*BMP4*	Bone morphogenetic protein 4	NM_001101031.2	0.723	0.0003
*CACNB2*	Calcium channel voltage dependent beta 2	XM_003482816.1	0.72	0.0002
*COL8A1*	Collagen, type VIII, alpha 1	XM_001926443.3	0.6	< 0.0001
*DCT*	Dopachrome tautomerase	NM_001025227.1	0.7	0.007
*ITGAV*	Integrin, alpha 5	NM_001083932.1	0.49	< 0.0001
*LHX2*	Lim homeobox protein 2	NM_001170519.1	0.61	< 0.0001
*PMEL*	Premelanosome protein	XM_003481614.3	1.50	< 0.0001
*SLC16A12*	Solute carrier family 16, member 12	XM_001928811.2	0.64	< 0.0001
*SLC24A5*	Solute carrier family 24, member 5	XM_003121523.1	1.58	< 0.0001
*SMAD6*	SMAD family member 6	XM_003480446.1	0.69	< 0.0001
*TYR*	Tyrosinase	NM_001025212.1	0.36	< 0.0001

Relative expression (*2*^*-ΔΔCt*^) in cultured RPE cells was normalized by the expression in native RPE cells. GAPDH was used as housekeeping gene. Statistic analysis (GraphPad Prism, multiple *t-*test, Holm-Sidak method, with alpha = 1.000%, n = 3 biological triplicates).

### OTX2S binds to the chromatin of the *RDH10* gene

**Fig 7:**
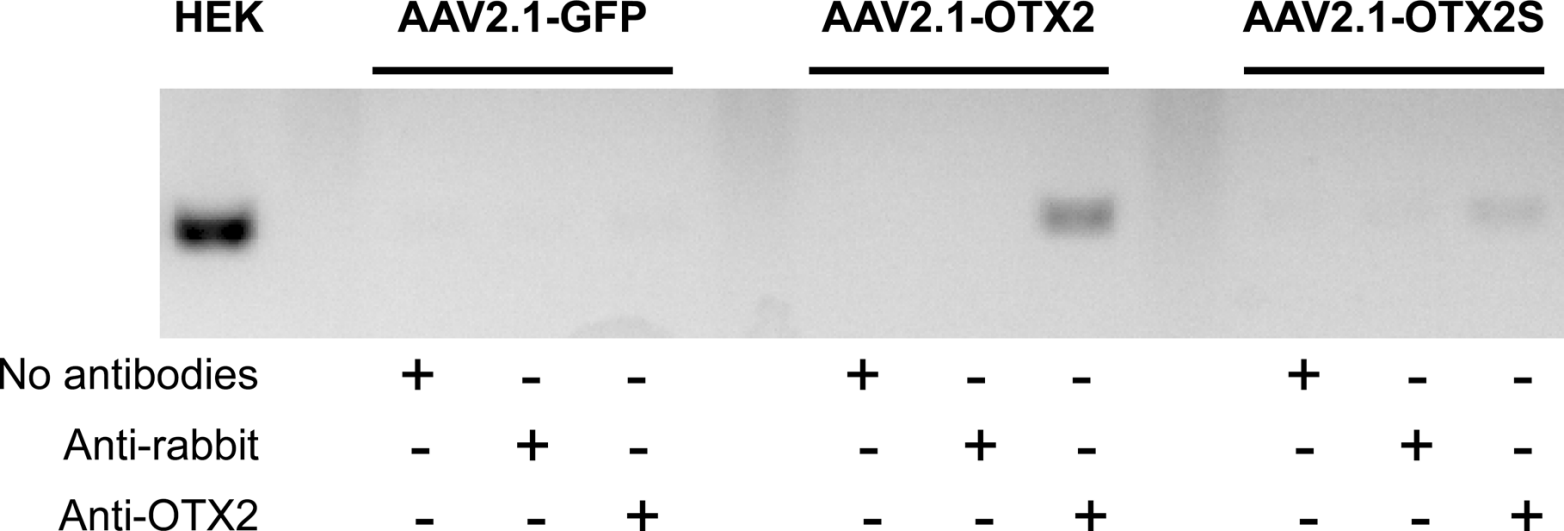
OTX2S interacts with the chromatin at the *RDH10* promoter. CHIP experiment performed with HEK293 cells infected with recombinant AAV2.1 vectors as indicated. Anti-rabbit and no antibodies are the negative controls. HEK represents the amplification product obtained with HEK293 DNA.

The mechanism by which OTX2S exerts transdominant activity was studied in HEK293 cells. Since pig RPE cells express endogenous OTX2 (**Fig 6D**), it is not possible to distinguish signal coming from OTX2S to that of endogenous OTX2. The problem we had to face is that HEK293 is a cell line derived from human embryonic kidney and does not express the tyrosinase gene. We looked at publically available data for the expression of gene regulated by OTX2 in HEK293 cells [57]. We found that *RDH10*, a gene recently found to be regulated by OTX2 in the RPE is expressed by HEK293 cells [58]. We infected HEK293 cells by AAV2.1 recombinant vectors; GFP, OTX2 or OTX2S. Eleven days after infection, the chromatin was crosslinked and immunoprecipitated with anti-OTX2 antibodies or negative control. The DNA was purified from the immunoprecipitated chromatin and amplified with primers located within the *RDH10* promoter. No product was amplified with cells traduced by AAV2.1-GFP confirming that those cells do not express OTX2 (**Fig 7**). The *RDH10* promoter was amplified in cells transduced by AAV2.1-OTX2 when the OTX2 was used but not the anti-rabbit antibodies showing that OTX2 expressed through the use of AAV infection is binding to the *RDH10* promoter. A similar PCR product of lower intensity was amplified in HEK293 cells transduced by AAV2.1-OTX2S when immunoprecipitated with anti-OTX2 antibodies. This demonstrates that OTX2S, an OTX2 splice variant with a truncation in the homeodomain is binding to the *RDH10* promoter. This binding is most probably indirect and might proceed through the interaction of OTX2S with DNA binding proteins bound directly to DNA elements within this promoter.

## Discussion

Mutations in genes encoding the splicing factors PRPF3, PRPF8, PRPF31, RP9, and SNRNP200 can cause non-syndromic *retinitis pigmentosa*, a severe form of inherited blindness leading to rod photoreceptor degeneration [7,9,59,60]. The mechanism by which these genetic defects affect rod photoreceptors specifically is still a mystery. In an effort to identify genes that might be affected by these splicing defects, we sequenced two normalized cDNA libraries representing 115,706 clones. We choose here to functionally characterize a novel splice variant of the *Otx2* gene, *Otx2S*. The novel protein OTX2S does not activate the tyrosinase promoter but exerts a transdominant activity in primary RPE cells as opposed to HEK293 cells. OTX2S interferes with the tyrosinase promoter modulating activity of both OTX2 and OTX2L. This RPE specific activity most likely results from the presence of tyrosinase promoter regulatory proteins in primary RPE cells, that are absent in HEK293 cells. OTX2S’s transdominant activity was also observed on endogenous OTX2 (**Fig 5B** and **Fig 6E**). The identity of the putative missing protein remains to be established. One possible candidate is MEIS2. MEIS2 physically interacts with OTX2 and competes with the Groucho co-repressor protein TLE4 for binding to OTX2, thereby releasing OTX2 from TLE4-mediated repression [61]. In the adult mouse, *Meis2* is expressed in the olfactory bulb, dorsal striatum, nucleus accumbens, neural retina and RPE (not shown). If OTX2S interacts also with MEIS2, it will potentially titrate the positive effect of endogenous MEIS2 on OTX2 activity via TLE4, and on *Tyr*-promoter activity. Since our collection did not include a canonical *Meis2* clone, we address the question of the mechanism of action of OTX2S by studying its interaction with the promoter of OTX2-regulated genes. We used HEK293 cells as they do not express OTX2 and showed by chromosomal immunoprecipitation that OTX2S as OTX2 binds the promoter of the *RDH10* gene. Because OTX2S lacks part of the homeodomain, its association may be indirect through physical association with an unknown protein bound to the *RDH10* promoter. It is not through homodimerization with OTX2 since the interaction does not require the co-infection of OTX2S with OTX2. This unknown protein must be expressed in HEK293 cells, so that its identity might not reveal the entire mechanism through which OTX2S exerts its transdominant activity on the *Tyr* promoter since it was not observed, at least using 0.5 μg of plasmid, after HEK293 transfection. This is also supported by the fact that the delivery of OTX2S in RPE cells does not lead to the reduction of *RDH10* expression (**Fig 6E**). We have demonstrated that OTX2S is able to associate to an OTX2-regulated promoter but more also further studies would reveal the way it acts of transcription activation.

What would be the role of OTX2S in the retina? Given its low level of expression, *Otx2S* represents only ~2% of that of *Otx2* in the retina and RPE, it seems unlikely that OTX2S titrates away a positive regulatory factor globally, in all RPE cells. In certain physiological circumstances, OTX2S might be expressed at a biologically significant level in a subpopulation of RPE cells. OTX2S, by its ability to counteract the positive effect of OTX2 on tyrosinase expression, may limit terminal differentiation of that putative subpopulation of RPE cells in which it is more highly expressed. In embryonic chick and mouse, RPE can transdifferentiate into neural retina, a property lost by mature RPE [62]. Apparently, a subpopulation of RPE cells in the adult conserves the capacity to proliferate *in vitro* [63]. We could speculate that these cells express a sufficient amount of OTX2S to maintain a certain degree of plasticity. Alternatively, RPE cells are subject to epithelial to mesenchymal transition *in vitro* or following retinal detachment [64,65]. We reasoned that OTX2S may be involved in the early phase of this pathological process by blocking the effect of OTX2 on RPE differentiation. But the change in expression of *Otx2S* and *Otx2* in a model of retinal detachment in the rat did not differ in polarity (**Fig 2E**), so it is difficult with those results to conclude.

The above-described identification and analysis of *Otx2* represents an approach that could be used to investigate other transcription factor isoforms, such as the novel variants of the four other transcription factors revealed by this project (**S6 Fig**). This and the fact that the dataset was generated by Sanger sequencing motivates us to conserve this unique EST collection as glycerol stocks, which are available to the international community of retinal biologists, and to develop an interactive repository, RetinaDB, which will provide ongoing annotation of the clones in the collection.

## Supporting Information

S1 Fig Identification of clones in the collection. (A) The acronym corresponds to LA: project name, 0 or 1: rearrangement code, A or B: plate set, AA: library name, NNN: plate number, X or Z: plate format, XXX: plate well, CMX: sequencing primer. (B) Library display in RenitaDB (http://kbass.institut-vision.org/KBaSS/dna/dnaform.php) and internal and external links. The technical form indicates the position of clone in the collection kept at -80C.
(PDF)

S2 Fig Protocol used to identify splice junctions specific to mouse and rat libraries compared to the human splice junction library. Definition of the splice junction. Schematic representation of a potential gene composed of 3 exons. Splice junction sequence is composed of the 50 bases of the 3’ part of an exon and the 50 bases of the 5’ part of the following exon excluding the exons overlapping 5’ and 3’ UTRs.
(PDF)

S3 Fig Transduction efficiency in AAV transduced- versus transiently transfected-RPE cells. The expression of the transgene in RPE cells started at day 4 and increased with a maximum at day 10. The expression of the transgene in lipofectamine transduction is not stable and after day 4 no GFP expression was further detected.
(PDF)

S4 Fig Absence of promoter competition. No change in the expression of the non-relevant promoter TK-Renilla luciferase was observed. (n = 4, ANOVA Holm-Sidak's multiple comparisons test).
(PDF)

S5 Fig Expression of the *Otx2* splicing variants. DNA sequence alignment of the *Otx2* splicing variants region corresponding to the homeodomain. P1 and P6 correspond to specific primers used for the amplification of different variants.
(PDF)

S6 Fig Validation of the expression of *Rax* splicing variants. (A) DNA sequence alignment of the *Rax* splicing variants region corresponding to the clones found in our library. (B) Amplification of each splice variant using primers amplifying the region between exon 1 and 3. * Corresponds to reference sequence encoding for RAX protein.
(PDF)

S1 Table Primers used for gene screening by quantitative RT-PCR. (DOCX)
