# Supplementary material for: Identification of an Alternative Splicing Product of the Otx2 Gene Expressed in the Neural Retina and Retinal Pigmented Epithelial Cells
Source: PLoS One. 2016 Mar 17;11(3):e0150758. doi: 10.1371/journal.pone.0150758 (PMC4795653; doi:10.1371/journal.pone.0150758)
Supplement: S1 Fig — (A) The acronym corresponds to LA: project name, 0 or 1: rearrangement code, A or B: plate set, AA: library name, NNN: plate number, X or Z: plate format, XXX: plate well, CMX: sequencing primer. (B) Library display in RenitaDB (http://kbass.institut-vision.org/KBaSS/dna/dnaform.php) and internal and external links. The technical form indicates the position of clone in the collection kept at -80C. (PDF) [file pone.0150758.s001.pdf]

A.

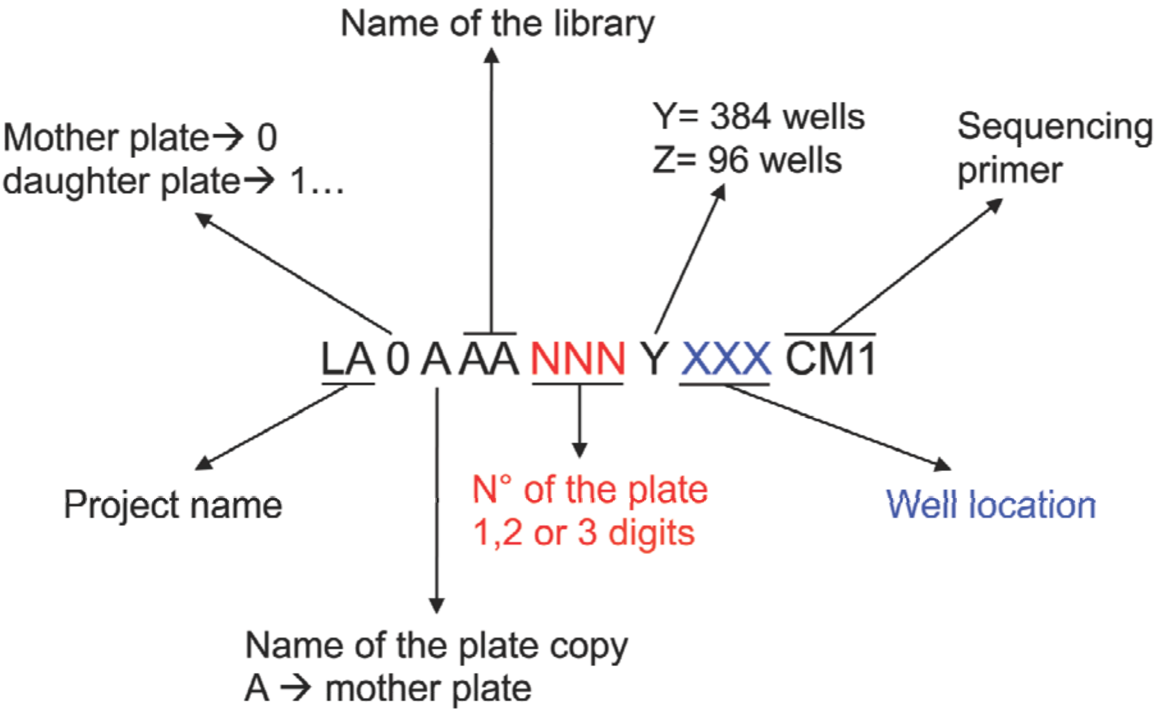

B.

Cluster: 10296 Varsplice: 1

Number	Sequence Name	Sequence Length	Technical Information	PFAM	UCSC	ATG Position	Coding Length	Clone Sequence	Protein Sequence
1	LA0ACA45YB14CM1	1076	Technical Form			no		Display	Display
2	LA0ACA63YE20CM1	1406	Technical Form	PF00046: 39-82,PF03529: 140-222	1-UCSC browser	344	1062	Display	Display
3	LA0ACA99YB24CM1	995	Technical Form			no		Display	Display
