# Supplementary figures and images for: Identification of an Alternative Splicing Product of the Otx2 Gene Expressed in the Neural Retina and Retinal Pigmented Epithelial Cells
Source: PLoS One. 2016 Mar 17;11(3):e0150758. doi: 10.1371/journal.pone.0150758 (PMC4795653; doi:10.1371/journal.pone.0150758)

A.

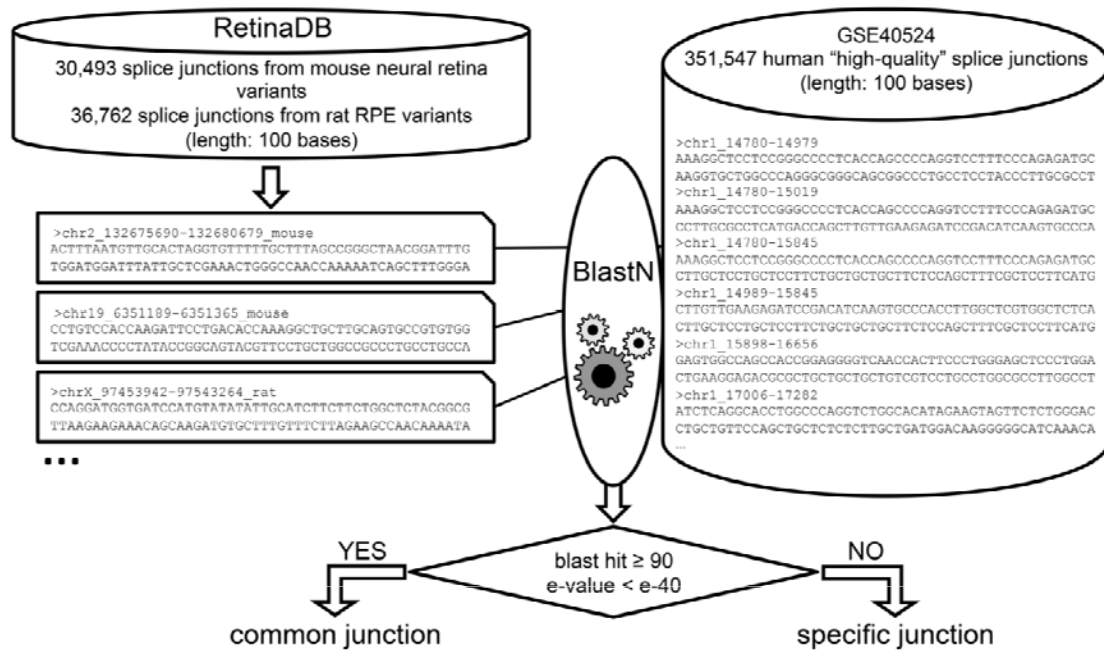

D.

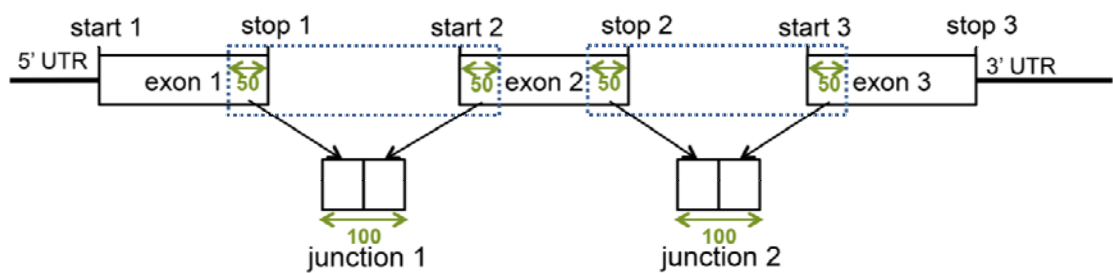

Supplement: S2 Fig — Definition of the splice junction. Schematic representation of a potential gene composed of 3 exons. Splice junction sequence is composed of the 50 bases of the 3’ part of an exon and the 50 bases of the 5’ part of the following exon excluding the exons overlapping 5’ and 3’ UTRs. (PDF) [file pone.0150758.s002.pdf]

AAV2.1-GFP (DAY 12)

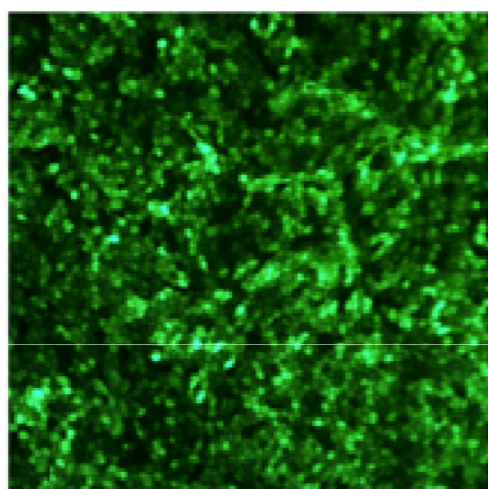

Lipofectain transfection (DAY 3)  
peGFP-C1

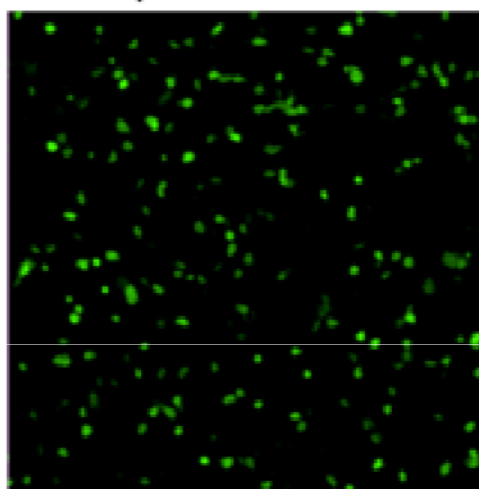

Supplement: S3 Fig — The expression of the transgene in RPE cells started at day 4 and increased with a maximum at day 10. The expression of the transgene in lipofectamine transduction is not stable and after day 4 no GFP expression was further detected. (PDF) [file pone.0150758.s003.pdf]

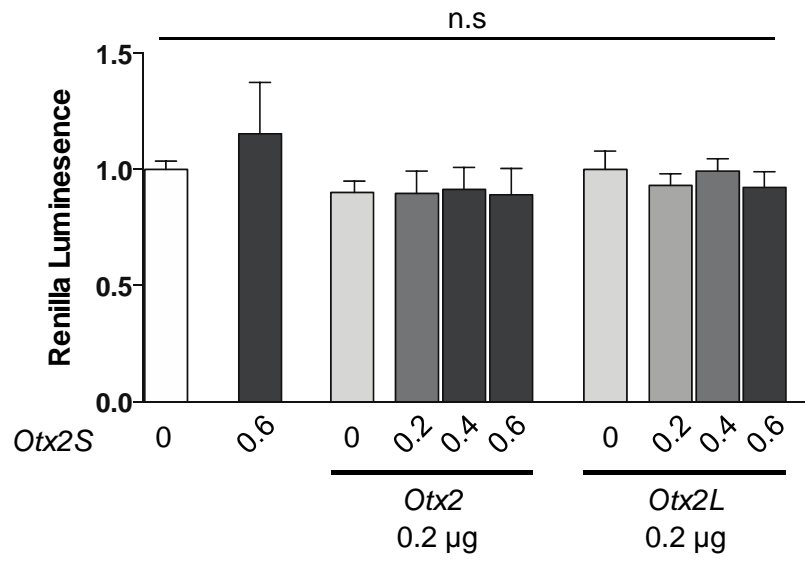

Supplement: S4 Fig — No change in the expression of the non-relevant promoter TK-Renilla luciferase was observed. (n = 4, ANOVA Holm-Sidak's multiple comparisons test). (PDF) [file pone.0150758.s004.pdf]

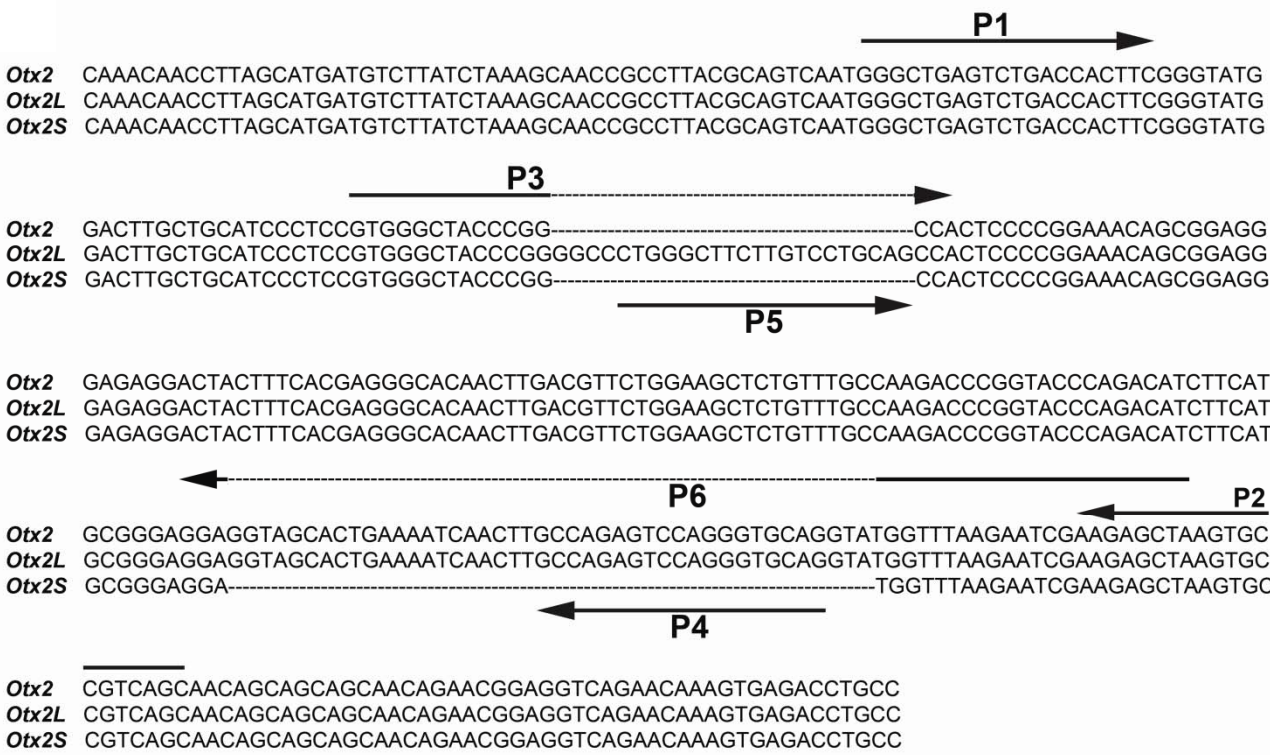

Supplement: S5 Fig — DNA sequence alignment of the Otx2 splicing variants region corresponding to the homeodomain. P1 and P6 correspond to specific primers used for the amplification of different variants. (PDF) [file pone.0150758.s005.pdf]

A.

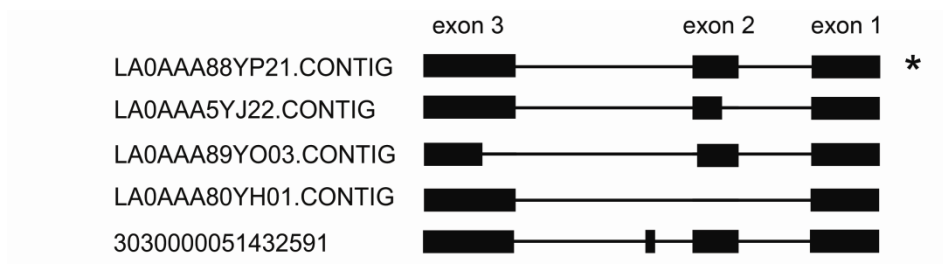

B.

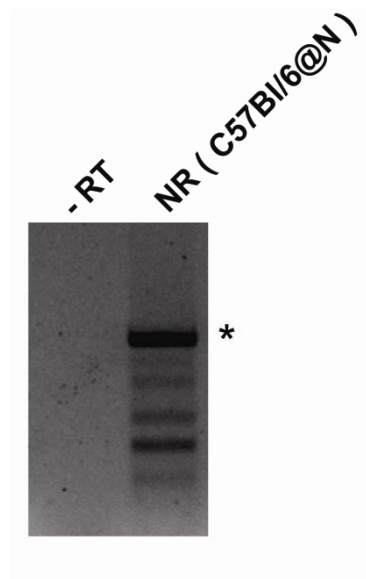

Supplement: S6 Fig — (A) DNA sequence alignment of the Rax splicing variants region corresponding to the clones found in our library. (B) Amplification of each splice variant using primers amplifying the region between exon 1 and 3. * Corresponds to reference sequence encoding for RAX protein. (PDF) [file pone.0150758.s006.pdf]
