# Supplementary material for: Identification of an Alternative Splicing Product of the Otx2 Gene Expressed in the Neural Retina and Retinal Pigmented Epithelial Cells
Source: PLoS One. 2016 Mar 17;11(3):e0150758. doi: 10.1371/journal.pone.0150758 (PMC4795653; doi:10.1371/journal.pone.0150758)
Supplement: S1 Table — (DOCX) [file pone.0150758.s007.docx]

**Table S1. Specific primer sequence used for quantitative RT-PCR**

| **Gene Name** | **NCBI**  **Accession No** | **Specie** | **PRIMERS (5’ > 3’)** | |
| --- | --- | --- | --- | --- |
|  |  |  | Forward | Reverse |
| *BDNF* | NM_214259.2 | Pig | CCTTTGGAGCCTCCTCTTCT | CCTCATGGACATGTTTGCAG |
| *BEST1* | XM_003353833.1 | Pig | CCAAAGACCAGAGCCTTCAG | GACAGATCGGCCAGGTTAAA |
| *BMP4* | NM_001101031.2 | Pig | GGCTGGAATGACTGGATTGT | AGTTGACCAGGGTCTGAACG |
| *CACNB2* | XM_003482816.1 | Pig | CATGAACAGAGAGCCAAGCA | GGAGGTGTCGATTTTCTGGA |
| *CRX* | XM_003127265.1 | Pig | CTGCCTCTGCTTTCTGCTCT | GGATCCAGGCCACTGAAATA |
| *CLDN19* | NM_001160084.1 | Pig | CATGGTCCTCAGTGTTGTCG | AGGGTGGCATACCATGAGAC |
| *CNTF* | XM_003122707.2 | Pig | AACCTGGACTCTGTGGATGG | ACGGTAAGCTCGGAGGTTCT |
| *COL8A1* | XM_001926443.3 | Pig | CCACCTCAAATTCCTCCTCA | CCGGCTGAATTTCCTTCATA |
| *CRYAB* | XM_003357294.1 | Pig | CGAGGAACTCAAGGTCAAGG | AGGACCCCATCAGATGACAG |
| *CTSD* | NM_001037721.1 | Pig | ATCAAGGTCGAGAGGCAGAC | TCTGCTGCATCAGGTTATCG |
| *DCT* | NM_001025227.1 | Pig | ACAGCGAGAAACTGCCAAGT | AAGGCATTCCTGAAGCTGAA |
| *FADS1* | NM_001113041.1 | Pig | CTATGTGCGCATCTTCCTCA | CTGTGTCACCCACACAAACC |
| *GAPDH* | NM_001206359.1 | Pig | ACCCAGAAGACTGTGGATGG | AAGCAGGGATGATGTTCTGG |
| *GPNMB* | NM_001098584.1 | Pig | GATTCATGATCCCAGCCACT | TTGCACAGTGAGGTTGAAGC |
| *ITGAV* | NM_001083932.1 | Pig | GTTCTGCTTAAAGGCGGATG | GTCGAATTGCTCCTTTCTGC |
| *ITGB5* | NM_001246669.1 | Pig | TGCAGCACCAAGAGAGATTG | GTTTTGGCAGGTCTGGTTGT |
| *ITGB8* | NM_001097424.1 | Pig | GCCTGGGTGTTTTCAGTTGT | TTGAGCACACCATCCACATT |
| *KCNJ13* | XM_001926506.3 | Pig | TGTCCACTGGCTTGTCTTTG | GGAGAAGGCAGCTGTGAAAC |
| *KRT8* | NM_001159615.1 | Pig | GCATGAGTCTGGTTGGAGGT | AGCAGGCTCTGGTTCACTGT |
| *KRT18* | XM_003126180.3 | Pig | ACCTCAGGACCTCAGCAAGA | GTGTCATCTCAGCAGCTCCA |
| *LHX2* | NM_001170519.1 | Pig | AGTTCAGGCGCAACCTCTTA | GCAGGGTGGGACTAGTCAAG |
| *LRAT* | NM_001244920.1 | Pig | AGTGTTCTTGCTTCGGCAGT | TTAGCCAGCCATCCATAAGC |
| *LRP8* | NM_001199891.1 | Pig | AAGGAGTGCGAAGAGAACCA | TCACAGGTGAAGTCGCTGTC |
| *MERTK* | XM_003124812.3 | Pig | TCCTGGAAAGTCTCCCTGAA | CCGGAAACTGTGTGTTGATG |
| *MITF* | NM_001038001.1 | Pig | GCCAATCGGCATTTGTTACT | GGATGAGGGAAAGTCCATGA |
| *MKI67* | NM_001101827.1 | Pig | CCTCGAAATTCCAGAAAGCA | GCCAAGGCCTCTTTCTTCTT |
| *OTX2* | XM_003353491.1 | Pig | AGAGGTCCTACCCCATGACC | AGTAGGAAGTCGAGCCAGCA |
| *PMEL* | XM_003481626.1 | Pig | AACCCAATGCTACCTGCATC | CCAGGCCTTCCAGACATAAA |
| *RDH10* | XM_001928082.3 | Pig | AAGGCTTTTCTTCCCACCAT | GGCGCAGTAATCCTCAACTC |
| *RPE65* | XM_003127931.2 | Pig | AGGCTGACACAGGCAAGAAT | AGCCAGATGGTCTCGTCACT |
| *SERPINF1* | NM_001078662.1 | Pig | GCTTACTTCAAGGGGCAGTG | AGAATCCAAGCCGTAGCGTA |
| *SLC16A3* | XM_003357925.1 | Pig | TACCTCACCACTGGGGTCAT | TAGCGGTTGAGCATGATGAG |
| *SLC16A8* | XM_003126028.1 | Pig | CAAGGCAGTGAGCGTTTTCT | CCAGGCTGTGTCACTGTAGC |
| *SLC16A12* | XM_001928811.2 | Pig | CTATGCACAGACAGCCTGGA | TGACAACACTCCCAAGTGGA |
| *SLC24A5* | XM_003121523.1 | Pig | TCTCCATCATCGGAGTTTCC | CAAGGCCATGAGCATGTAGA |
| *SLC39A12* | XM_003130728.1 | Pig | ACGGAAAACCCCTCAACTCT | TTGCCTTCCATACGTTCTCC |
| *SMAD6* | XM_003480446.1 | Pig | CACCCCATCTTCGTCAACTC | CGAAGTCGAACACCTTGATG |
| *TRPM3* | XM_001925032.3 | Pig | AGGAGACCTTGTCCCCAACT | TGCAGGAGCTTTGGATTCTT |
| *TYR* | NM_001025212.1 | Pig | ATGGAACCATGTCCCAGGTA | CATTGGCTTCCGGATACACT |
| *TYRP1* | NM_001025226.1 | Pig | TGTCTGATTCGTGCCAGAAG | GTGAGGAGAGGCTGATTTGC |
